# CI431, an Aqueous Compound from *Ciona intestinalis L.*, Induces Apoptosis through a Mitochondria-Mediated Pathway in Human Hepatocellular Carcinoma Cells

**DOI:** 10.1155/2011/292873

**Published:** 2011-07-21

**Authors:** Linyou Cheng, Ming Liu, Cuicui Wang, Haizhou Liu, Yuyan Zhang, Xiukun Lin

**Affiliations:** ^1^Institute of Oceanology, Chinese Academy of Sciences, Qingdao 266071, China; ^2^Graduate University, Chinese Academy of Sciences, Beijing 100049, China; ^3^College of Chemical Engineering, Qingdao University of Science and Technology, Qingdao 266042, China

## Abstract

In the present studies, a novel compound with potent anti-tumor activity from *Ciona intestinalis L.* was purified by acetone fractionation, ultrafiltration, gel chromatography and High Performance Liquid Chromatography. The molecular weight of the highly purified compound, designated CI431, was 431Da as determined by HPLC-MS analysis. CI431 exhibited significant cytotoxicity to several cancer cell types. However, only a slight inhibitory effect was found when treating the benign human liver cell line BEL-7702 with the compound. To explore its mechanism against hepatocellular carcinoma, BEL-7402 cells were treated with CI431 in vitro. We found that CI431 induced apoptotic death in BEL-7402 cells in a dose- and time-dependent manner. Cell cycle analysis demonstrated that CI431 caused cell cycle arrest at the G2/M phase, and a sub-G1 peak appeared after 24 h. The mitochondrial-mediated pathway was implicated in this CI431-induced apoptosis as evidenced by the disruption of mitochondrial membrane potential. The results suggest that the CI431 induces apoptosis in BEL-7402 human hepatoma cells by intrinsic mitochondrial pathway.

## 1. Introduction

It is now clear that the oceans are not only home to a tremendous diversity of species but that their inhabitants produce also a wealth of natural products [1]. Since the 1950s, many structurally diverse natural products with astounding bioactivities have been discovered from marine organisms [2]. These compounds are mainly isolated from sessile animals, such as sponges, tunicates, corals, mollusks, and bryozoans [3, 4]. 

Among sessile animals, tunicates have received the most attention. More commonly known as Ascidiacea, members of the class Ascidiacea (Ascidians) are the most highly investigated tunicates, since they present a benthonic stage in their life, making their collection easier. The chemistry of ascidians has become one of the most active fields of marine natural products; it has been amply demonstrated that these sea creatures are prolific producers of unusual structures with significant bioactivities. Most of these products fall within the area of cancer therapy [5], and a significant number of ascidian-derived compounds have entered into preclinical and clinical trials as antitumor agents [3, 6]. 

Didemnin B, is perhaps the most studied marine natural product. This cyclic peptide was isolated from the Caribbean tunicate Trididemnum solidum [7]. Early investigation into the bioactivity of this compound revealed its strong antiproliferative effects in vitro against a variety of human tumor cell lines. It was developed by NCI and went through phase II clinical trials but was withdrawn because it proved to be too toxic. Although didemnin B was never carried into Phase III trials, activity focused on developing the compound as a potential cancer treatment helped pave the way for the rest of the marine-derived products following it into the development pipeline. Aplidine [8], Vitilevuamide [9], Diazonamide [10], and ET-743 [11], all of them were compounds with efficient antitumor activity isolated from Ascidians. 

Hepatocellular carcinoma (HCC) is the fifth most common cancer [12], with a 5-year survival rate of less than 5% and is the fourth leading cause of cancer death worldwide [13–15]. Its incidence has been increasing over the past few decades in some areas such as Europe, USA, and East Asia [16, 17]. Despite the high mortality and frequency of this cancer, surgical resection is an available option for only a small proportion of patients because metastases are often present when the cancer is discovered. In addition, because of the inherent chemotherapy-resistant nature of HCC, systemic cytotoxic chemotherapy agents are minimally effective at improving patient survival [18]. Thus, novel strategies and agents, which have greater targeting on HCC but lower toxicity for normal liver cells, are seen as a direction of enormous potential. 

Previous studies have shown that several agents derived from Ascidiae can induce the apoptosis of many cancer cells [5]. However, *Ciona intestinalis L.,* a selected species in the present study, has not been studied for its anticancer effects. Therefore, we attempted to investigate the growth-inhibitory and apoptotic effects of components from *Ciona intestinalis L.* against human liver cancer cells. A bioguided isolation was performed to purify the active components from the species. We found that a component, CI431, was a potent inhibitor against human hepatoma Bel-7402 cells and may be developed as a novel class of anticancer agents. 

## 2. Subjects and Methods

### 2.1. Materials

*Ciona intestinalis L.* was obtained from the Xunshan Fishery Company of Rongcheng, China. The animals were identified by professor Fuhua-li at Institute of Oceanology, Chinese Academy of Sciences. The human hepatocellular cancer BEL-7402, human colorectal cancer HCT116, human cervical cancer Hela cells as well as human lung adenocarcinoma A549, breast cancer MCF-7, and human benign liver cell BEL-7702 cells were obtained from American Type Culture Collection. 

### 2.2. Extraction and Purification of the Compound from *Ciona intestinalis L.*

Briefly, 50 kg fresh *C. intestinalis *were smashed and incubated at 70°C for 30 min and were then centrifuged at 8,000 rpm for 30 min. Next, the supernatants were added to 3 volumes of acetone at 4°C for 12 h and were centrifuged at 10,000 rpm for 30 min once more. The acetone supernatants were collected and then were disposed through a 5 kDa ultrafiltration membrane (Millipore, USA). The residue with size <5 kDa was lyophilized and dissolved in a small amount of distilled water. The prepared extraction solution was applied to a Sephadex-G25 column and eluted with distilled water.

All of the fractions were collected and analyzed for cytotoxicity by MTT assay. The active fraction was lyophilized and loaded to a P2 Gel (Bio-Rad Laboratories, Inc) column. The column was eluted with distilled water. The active fraction was pooled and subjected to reverse-phase liquid chromatography on a C18 column (Agilent C18 4.6 × 250 mm) using an Agilent apparatus. The chromatography was performed at a flow rate of 0.5 mL/min, using 0.1% TFA as solvent A and 99.9% acetonitrile containing 0.1% TFA as solvent B. The gradient was 10–60% of solvent B for 50 min. The elution profile was monitored by online measurement of the absorbance at 214 and 280 nm. The fractions were pooled and freeze dried. All of the fractions were collected and analyzed by MTT assay. 

### 2.3. Cell Culture

The carcinoma cells BEL-7402, Hela, HCT116, and benign liver cell BEL-7702 were cultured in RPMI-1640 medium. The MCF-7 cell lines were cultured in DMEM medium. The A549 cell lines were cultured in F12 medium. All cells were incubated in media supplemented with 10% fetal bovine serum in a humidified atmosphere containing 5% CO_2_ at 37°C. The media and sera were purchased from Sigma Chemical.

### 2.4. Assessment of Cytotoxicity

The inhibitory effects of the antitumor agents on the growth of cancer cells as well as benign cells BEL-7702 were assessed in vitro by MTT assay [19]. Four thousand cells per well were seeded into a 96-well microplate. Cells were cultured in 180 *μ*L media of RPMI-1640, 12 K, or DMEM for 24 h. The purified compound with different concentrations was added to the medium. After 48 h, MTT solution (50 *μ*L, 0.5 mg/mL) was added into each well, and the cells were incubated for another 4 h. After adding 150 *μ*L DMSO to each aspirated well, the plate was gently agitated until the color reaction was uniform. The OD590 was determined by a microplate reader (Bio-Tek Instruments, USA) with subtraction of background absorbance [20].

### 2.5. CI431-Induced Morphological Changes of BEL-7402 Cells

Morphological alterations of BEL-7402 cells after CI431 treatment were investigated using phase contrast microscopy and SEM. The cells were seeded and cultured in 96-well plates, as described above. After incubation with 50 *μ*g/mL CI431, the morphology of cells was observed under the CKX41 phase contrast microscopy (Olympus, Japan) and photographed at 0, 12, and 24 h, respectively. In the SEM experiment, BEL-7402 cells were grown onto poly-L-lysine-coated coverslips in 6-well plates for 24 h to allow firm attachment. Then, they were treated with 50 *μ*g/mL CI431 and incubated for 0, 12, and 24 h. The medium containing CI431 was removed, and subsequently the cells were fixed in glutaraldehyde. After fixation overnight at 4°C, the coverslip was dehydrated in ethanol and dried in a critical point dryer. Cells on coverslip were coated with gold and analyzed by the S-3400N SEM (Hitachi, Japan).

### 2.6. DAPI (4′-6-diamidino-2-phenylindole) Staining

The cells BEL-7402 were grown onto poly-L-lysine-coated coverslips in 6-well plates and treated with CI431 as described above for the SEM assay. Then the medium containing CI431 was removed, and subsequently the cells were fixed in paraformaldehyde. After fixation overnight at 4°C, the coverslips were stained with DAPI solution followed by observation with a fluorescence microscope [21].

### 2.7. Flow Cytometric Analysis

The cells BEL-7402 were incubated with 20, 40, and 80 *μ*g/mL of CI431 for 24 h. The cells were harvested and fixed in ice-cold 70% (v/v) ethanol for 24 h at 4°C. After centrifugation, the cell pellet was resuspended in PBS; the cells were subjected to propidium iodide (PI) staining and then analyzed by a flow cytometry (FACSCalibur; BD Biosciences, USA) [22].

### 2.8. Mitochondrial-Membrane Potential (Δ*ψ*m)

The JC-1 Mitochondrial Apoptosis Detection Kit was used to detect Δ**ψ*m* disruption. JC-1 is selectively accumulated within intact mitochondria to form multimer J-aggregates emitting fluorescence light at 590 nm (red) at a higher membrane potential, and monomeric JC-1 emits light at 527 nm (green) at a low membrane potential. Thus, the fluorescence color of JC-1 represents mitochondrial-membrane potential, which can be analyzed by FACS system [23]. According to the manufacturer's protocols, cells were seeded in 12-well plates at a density of 3 × 10^5^ cells/mL and treated with CI431 at doses of 20, 40, and 80 *μ*g/mL for 24 h. After treatment with CI431 200 *μ*L, prewarmed incubation buffer containing 0.2 *μ*L MitoCapture was added to each well and plates were incubated for 15 min at 37°C in a 5% CO_2_ incubator. Then, cells were analyzed by a fluorescence spectrophotometer (F-4500, HITACHI).

### 2.9. Statistical Analysis

All experiments were done three times in triplicate (*n* = 9), and the results were expressed as means ± SD (standard deviation). A one-way analysis of variance (ANOVA) and the Duncan test were used for multiple comparisons (SPSS program, ver 10.0).

## 3. Results

### 3.1. Extraction and Purification of the Compound from *Ciona intestinalis L.*

In order to isolate novel antitumor agents from *Ciona intestinalis L.*, we developed a purification protocol involving heat-inactivation, acetone precipitation, ultrafiltration concentration, gel filtration, and reverse-phase chromatography HPCL. Briefly, acetone precipitation, and ultrafiltration concentration were carried out to obtain a pool of antitumor agents as previously described. The fraction obtained after acetone precipitation was concentrated through a 5 kDa ultrafiltration membrane. The fraction of acetone precipitation solution, which contained several major compounds below 5 kDa, showed strong inhibitory activity against several tumor cell lines.

**Figure 1:**
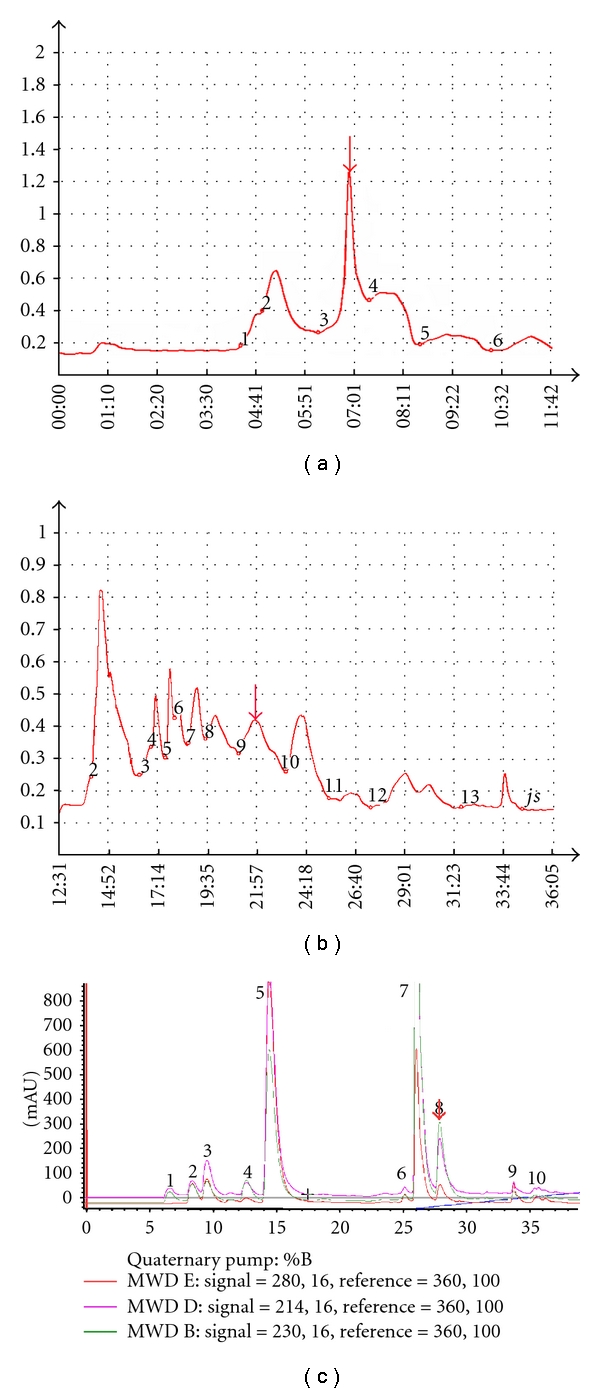
Elution patterns on column chromatography. The fraction of acetone precipitation was applied to Sephadex G25 column (a). The active fraction from Sephadex G25 chromatography was applied to P_2_ column (b). The active fraction from P_2_ was further purified by high-performance liquid chromatography (c). The arrow indicates the active component during the purification.

The fraction of acetone precipitation compounds was applied to a Sephadex-G25 column and eluted with distilled water (Figure 1(a)). The active fractions were further purified by P2 column (Figure 1(b)). After gel chromatography, the active components were submitted to reverse-phase chromatography using a C18 column, and purified to homogeneity (Figure 1(c)). The purified agent, designated CI431, was revealed as a single MW by MS. 

### 3.2. Assessment of Cytotoxicity

**Figure 2:**
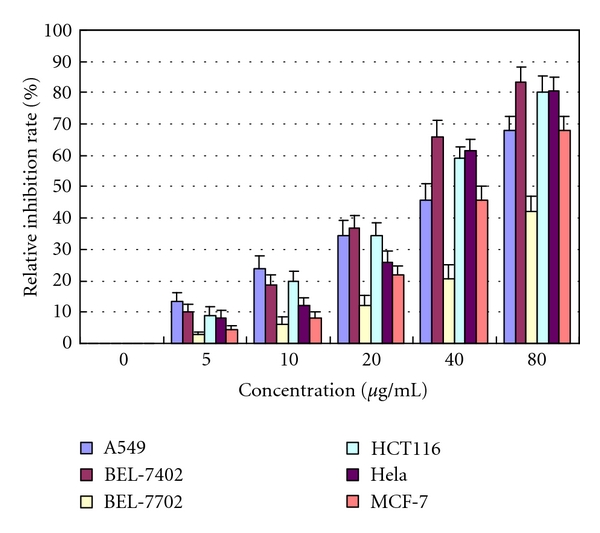
CI431 inhibited the growth of several types of cancer cells. Human hepatoma BEL-7402, human colon cancer HCT116, human breast cancer MCF-7, human cervical cancer Hela cells, and human lung adenocarcinoma A549 as well as human benign liver cell BEL-7702 cells were incubated in the absence or presence of certain concentrations of CI431 for 48 h at 37°C. MTT assay was performed to determine the growth inhibition of different cancer cells and benign BEL-7702 cells by CI431. The experiments were performed more than three times.

MTT assays were performed to investigate the effects of CI431 on the proliferation of six cell lines. As shown in Figure 2, CI431 (over 50 *μ*g/mL) had a significant growth-inhibiting effect on the five cancer cell lines and a slight growth-inhibiting effect on the benign liver BEL-7702 cell line. Results indicated that CI431 had a significant grow-inhibiting effect on the five cell lines in a time-dependent manner (data not shown) and in a dose-dependent manner (Figure 2). Among these cell lines, the BEL-7402 cells were much more sensitive than the other cell lines. From this result, BEL-7402 cells were chosen for the subsequent experiments. 

### 3.3. CI431-Induced Morphological Changes of BEL-7402 Cells

**Figure 3:**
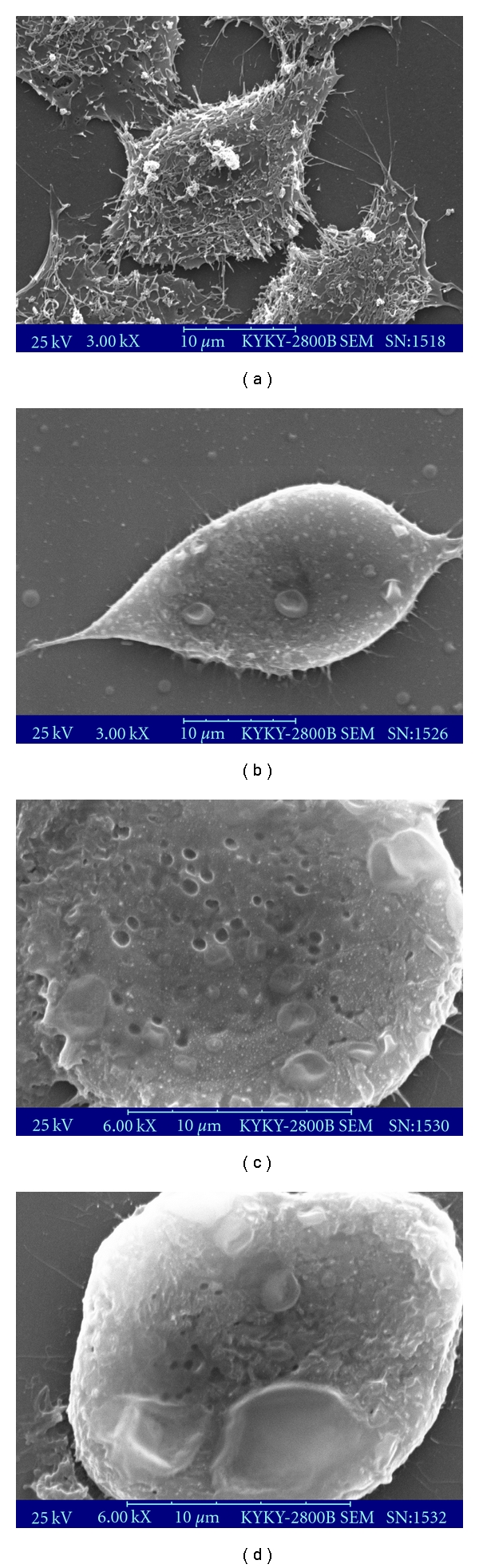
Morphological analysis of BEL-7402 cells induced by CI431. BEL-7402 cells were grown on poly-L-lysine-coated coverslips for 24 h to allow firm attachment and treated with 50 *μ*g/mL CI431 for certain time intervals. Cells were fixed on coverslips coated with gold and analyzed by using the KYKY-2800B SEM. The cells were untreated (a) or treated with CI431 for 12 (b), 18 (c), and 24 h (d). The untreated BEL-7402 cells showed a normal smooth surface. In contrast, the cells treated with CI431 became rounded, and the surface of the cell membrane was markedly disrupted (Scale bar = 10 *μ*m).

The morphologic changes of the cell membrane were clearly visualized by SEM. Remarkable alterations of the cell membrane of BEL-7402 cells were observed after CI431 treatment. The architecture of untreated BEL-7402 cells displayed a typical polygon shape (Figure 3(a)). However, the morphology of the cells started to change after incubation with CI431. The cells detached from the substratum, became spindle shape (Figure 3(b)), and separated from each other after exposure to CI431 for 12 h. Membrane bulge and detachment from cytoplasmic inclusion were observed in 18 h and 24 h after CI431 treatment (Figures 3(c) and 3(d)). The untreated BEL-7402 cells showed a normal smooth surface. In contrast, the cells treated with CI431 became rounded, and the surface of the cell membrane was markedly disrupted.

### 3.4. DAPI (4′-6-diamidino-2-phenylindole) Staining

**Figure 4:**
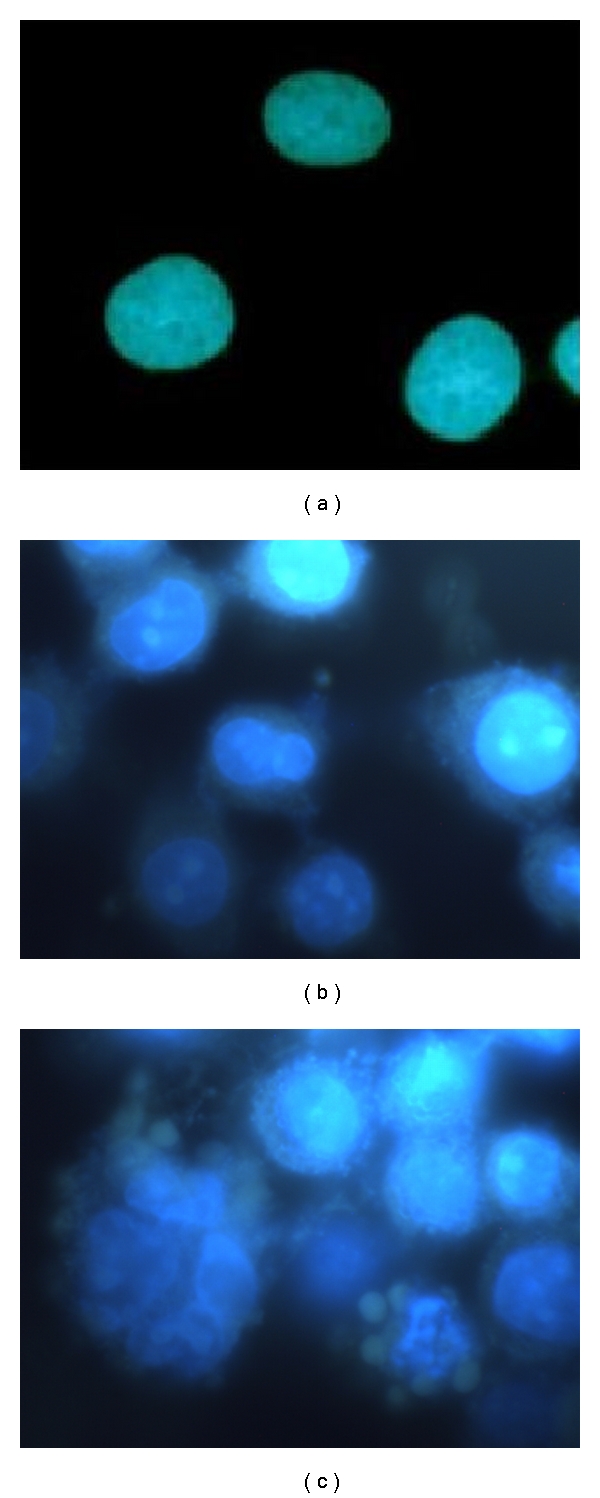
DAPI staining assay. The cells BEL-7402 were grown on poly-L-lysine-coated coverslips in 6-well plates and treated with CI431. After incubation for 24, the cells were stained with DAPI and then observed under the fluorescence microscopy. BEL-7401 cells in the control medium were stained homogeneously with DAPI (a), whereas treatment with CI431 led to marked chromatin condensation and nuclear fragmentation together with the appearance of small structures like apoptotic bodies. These observations indicated that the cells treated with CI431 entered apoptosis (b, c).

We characterized the changes in the nuclear morphology by staining with DAPI. BEL-7401 cells in the control medium were stained homogeneously with DAPI, whereas treatment with CI431 led to marked chromatin condensation and nuclear fragmentation together with the appearance of small structures like apoptotic bodies, a biochemical hallmark of apoptosis. The sizes of CI431-treated BEL-7402 cells increased as compared with those of untreated cells. Double nucleated cells or giant multinucleated cells can be easily found. These observations indicated that the cells treated with CI431 entered apoptosis (Figure 4).

### 3.5. CI431 Induces Apoptosis and G2/S Phase Arrest in BEL-7402 Cells

**Figure 5:**
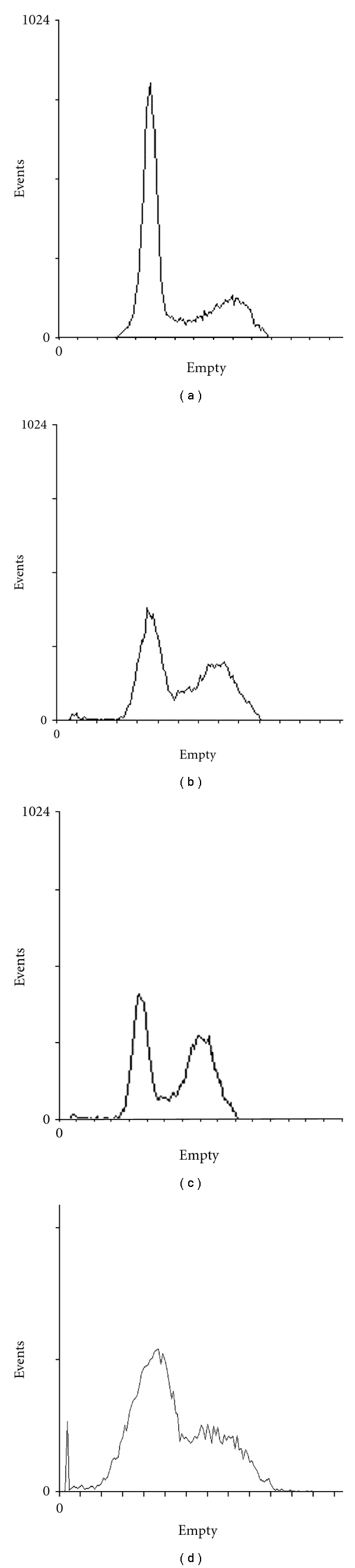
Effect of CI431 on cell cycle distribution. BEL-7402 cells were seeded at 3 x 10^4^/cm^2^ in 10-cm dishes and treated with CI431 at 20 (b), 40 (c) or 80 *μ*g/mL, respectively or without treatment (a). After incubation for 24 h, cells were collected and the DNAs were stained by PI, while the RNAs were removed by digestion with RNase A. The DNA contents of the cells were determined with the FACSCalibur cytometer. The results showed that CI431 significantly induced a G_2_/S phase arrest with a decrease in G_0_/G_1_ phase population at 20, 40, and 80 *μ*g/mL, while a dramatic increase of sub-G1 phase (hypodiploid cells) was observed at doses higher than 80 *μ*g/mL.

To gain an insight into the antiproliferation mechanism of CI431, the cell cycle distribution of CI431-treated cells was determined by flow cytometry analysis. The results showed that CI431 significantly induced a G_2_/S phase arrest with a decrease in G_0_/G_1_ phase population at 20, 40, and 80 *μ*g/mL, while a dramatic increase of sub-G1 phase (hypodiploid cells) was observed at doses higher than 80 *μ*g/mL (Figure 5).

### 3.6. Mitochondrial-Membrane Potential (Δ*ψ*m)

**Figure 6:**
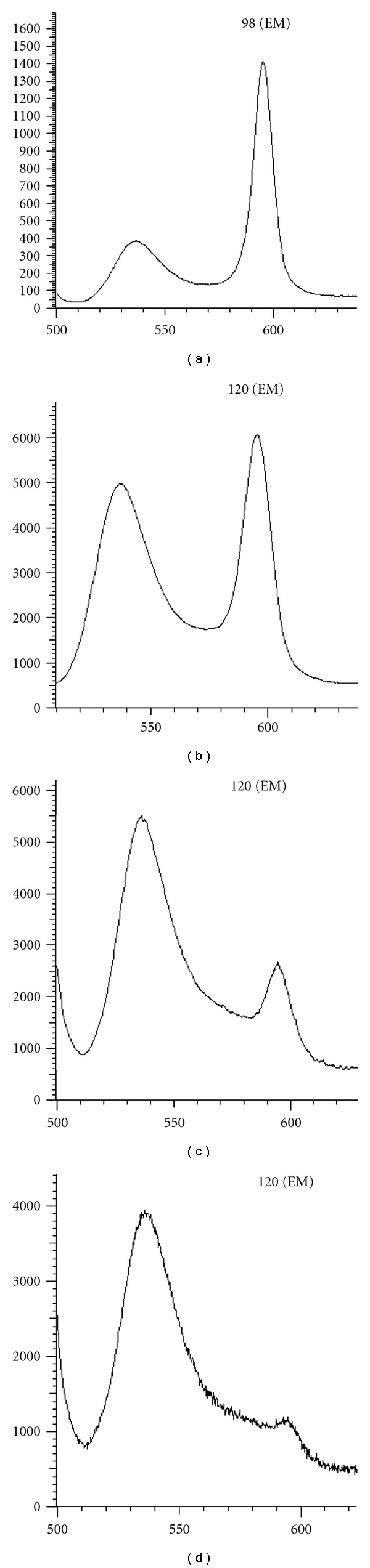
Cytofluorometric analysis of Δ**ψ*m *BEL-7402 cells were seeded in 12-well plates at a density of 3 × 10^5^ cells/mL and treated with CI431 at doses of 20, 40, and 80 *μ*g/mL for 24 h. After treatment with CI431 200 *μ*L pre-warmed incubation buffer containing 0.2 *μ*L MitoCapture was added to each well and plates were incubated for 15 min at 37°C in a 5% CO_2_ incubator. Then, cells were analyzed by a Fluorescence Spectrophotometer (F-4500, HITACHI, Japan). After treatment with CI431, Δ**ψ*m* began to decrease, and the ratio of green fluorescence intensity to red fluorescence intensity was 0.61 ± 0.07 (blank, a), 1.92 ± 0.19 (20 *μ*g/mL, b), 5.08 ± 0.45 (40 *μ*LgmL, c) and 8.28 ± 0.61 (80 *μ*g/mL, d), respectively, indicating disruption of mitochondrial function.

To investigate the involvement of the mitochondrial pathway, depolarization of the mitochondrion was analyzed by loading with JC-1. BEL-7402 were exposed to CI431 at doses of 20, 40, and 80 *μ*g/mL for 24 h, and the ratio of green fluorescence intensity to red fluorescence intensity was used for quantitative analysis of the disruption of Δ**ψ*m*. As shown in Figure 6, after treatment with CI431, Δ**ψ*m* began to decrease, and the ratio is 0.61 ± 0.07 (blank), 1.92 ± 0.19 (20 *μ*g/mL), 5.08 ± 0.45 (40 *μ*g/mL), and 8.28 ± 0.61 (80 *μ*g/mL), respectively, indicating disruption of mitochondrial function. 

## 4. Discussion

Over 17,000 biologically active compounds have been identified from marine sources, mainly isolated from sessile animals, such as sponges, tunicates, corals, mollusks, and bryozoans [3, 4]. Many of these substances are potent cytotoxins that are of great interest for anticancer drug development. The discovery of new marine drug candidates is a highly efficient process due to the availability of sophisticated screening, dereplication, and characterization techniques. In this study, we describe a novel protocol which allows efficient and rapid purification of low-molecular weight marine drug candidates with antitumor activity from* Ciona intestinalis L.* We have utilized for their isolation a combination of extraction/purification procedures, including heating, acetone fractionation, gel chromatography and high performance liquid chromatography. The proposed protocol resulted in obtaining homogeneous preparations of one compound from* Ciona intestinalis L.* with MW of 431 Da, which exhibited strong antitumor activity. The MW identity of the purified compound was determined by mass spectrometry. 

We used the MTT assay to test the cytotoxic effects of CI431 and found that CI431 exhibited potent cytotoxicity against various types of cancer cell lines in a dose- and time-dependent manner but inhibited the viability of Bel-7702, human benign liver cells, very slightly. That CI431 has opposite effects on tumor cells and normal cells is intriguing. This shows that CI431 may have little toxicity for normal liver cells. When the cells were stained with different concentrations of CI431 for 48 h, marked morphological changes were clearly observed, including chromatin condensation, nuclear and cytoplasmic fragmentation, and apoptotic body appearance. Further studies using PI staining and flow cytometry analysis showed that CI431 significantly induced a G2/S phase arrest with a decrease in G0/G1 phase population, and 17.3% hypodiploid cells were observed when the dose up to 60 *μ*g/mL. Considering figures of apoptosis bodies obtained from staining with DAPI and 17.3% hypodiploid BEL-7402 cells reserved from flow cytometry, we concluded that CI431 could induce the apoptosis of BEL-7402 cell lines. In addition, marked mitochondrial-membrane-potential changes were clearly observed especially after treatment with 0.1 mg/mL of CI431 for 24 h. Loss of mitochondrial-membrane potential (Δ*Ψm*) is an early event in apoptosis. It indicated that maybe CI431 induced BEL-7402 cell apoptosis through a mitochondria-mediated apoptosis pathway.

## 5. Conclusion

This study describes for the first time an efficient method for the purification of a kind of antitumor compound from *Ciona intestinalis L.* It could effectively induce apoptosis in HCC cell lines, mediated through a disruption of mitochondrial membrane potential. Our results suggest that CI431 is a promising drug candidate for the treatment of HCC. However, the specific mechanism of the apoptosis-inducing effect of CI431 has not yet been elucidated. The effective and powerful components in this extracted compound need to be further characterized. Because some secondary metabolites are generated in organisms just in a very short period of their life cycles, and some target chemicals are in low concntration, how to obtain enough chemicals for structure analyzing and preclinical testing has become a crucial problem in marine medicine research. In this study, the yield rate of CI431 from* Ciona intestinalis L.* was extremely low. In order to elucidate its structural formula, more samples should be collected, isolated, and purified.
